# Toward an Understanding of Disengagement from HIV Treatment and Care in Sub-Saharan Africa: A Qualitative Study

**DOI:** 10.1371/journal.pmed.1001369

**Published:** 2013-01-08

**Authors:** Norma C. Ware, Monique A. Wyatt, Elvin H. Geng, Sylvia F. Kaaya, Oche O. Agbaji, Winnie R. Muyindike, Guerino Chalamilla, Patricia A. Agaba

**Affiliations:** 1 Harvard Medical School, Boston, Massachusetts, United States of America; 2 University of California at San Francisco, San Francisco, California, United States of America; 3 Muhimbili University of Health and Allied Sciences, Dar es Salaam, Tanzania; 4 Jos University Teaching Hospital, Jos University, Jos, Nigeria; 5 Mbarara Regional Referral Hospital, Mbarara University of Science and Technology, Mbarara, Uganda; 6 Management and Development for Health, Dar es Salaam, Tanzania; St. Michael's Hospital, Canada

## Abstract

**Background:**

The rollout of antiretroviral therapy in sub-Saharan Africa has brought lifesaving treatment to millions of HIV-infected individuals. Treatment is lifelong, however, and to continue to benefit, patients must remain in care. Despite this, systematic investigations of retention have repeatedly documented high rates of loss to follow-up from HIV treatment programs. This paper introduces an explanation for missed clinic visits and subsequent disengagement among patients enrolled in HIV treatment and care programs in Africa.

**Methods and Findings:**

Eight-hundred-ninety patients enrolled in HIV treatment programs in Jos, Nigeria; Dar es Salaam, Tanzania; and Mbarara, Uganda who had extended absences from care were tracked for qualitative research interviews. Two-hundred-eighty-seven were located, and 91 took part in the study. Interview data were inductively analyzed to identify reasons for missed visits and to assemble them into a broader explanation of how missed visits may develop into disengagement. Findings reveal unintentional and intentional reasons for missing, along with reluctance to return to care following an absence. Disengagement is interpreted as a process through which missed visits and ensuing reluctance to return over time erode patients' subjective sense of connectedness to care.

**Conclusions:**

Missed visits are inevitable over a lifelong course of HIV care. Efforts to prevent missed clinic visits combined with moves to minimize barriers to re-entry into care are more likely than either approach alone to keep missed visits from turning into long-term disengagement.

*Please see later in the article for the Editors' Summary*

## Abstract 2

Norma Ware and colleagues conducted a large qualitative study among patients in HIV treatment programs in sub-Saharan Africa to investigate reasons for missed visits and provide an explanation for disengagement from care.

## Editors' Summary

**Background:**

The human immunodeficiency virus (HIV) infects cells of the immune system, destroying or impairing their function. As the infection progresses, the immune system becomes weaker, and the affected person becomes more susceptible to life-threatening infections. Over the past three decades, 25 million people have died from HIV, and according to the World Health Organization, in 2011, there were roughly 34.2 million people living with HIV, over 60% of whom lived in sub-Saharan Africa. Although HIV cannot be cured, the virus can be suppressed by combination antiretroviral therapy (ART) consisting of three or more antiretroviral drugs. ART controls viral replication and strengthens the immune system, allowing the affected person to fight off infections. With ART, HIV can be managed as a chronic disease: people living with HIV can live healthy lives as long as they take antiretroviral drugs regularly for the rest of their lives.

**Why Was This Study Done?:**

Unfortunately, poor retention in HIV programs is a huge problem: a large proportion—30%–60% in some settings in sub-Saharan Africa—of people starting ART, are lost to follow-up and stop taking treatment. Few studies have looked in depth at the reasons why people with HIV in sub-Saharan Africa miss clinic appointments or even stop coming altogether. So in this study in Tanzania, Uganda, and Nigeria, the researchers did a qualitative analysis from the patients' perspective on the reasons for missing clinic visits. Qualitative research can use information-gathering techniques, such as open-ended interviews, to develop an in-depth understanding of human behavior and the reasons behind such behavior.

**What Did the Researchers Do and Find?:**

The researchers indentified people to interview by using “tracking lists” from HIV/AIDS care clinics in the three countries—patient tracking by clinical trackers is increasingly used as a way to contact patients who have missed clinic appointments. The researchers included people in the study who had been tracked and contacted by clinic trackers, had been absent from the clinic for three months or more, and gave consent to be re-contacted by the researchers. The researchers interviewed participants, using their local language, on several topics, including their experiences of care at the clinic and of tracking, and the circumstances of missed appointments. The detailed accounts were transcribed, and then the researchers categorized the reasons for missing appointments into intentional and unintentional reasons.

Eight-hundred-ninety patients in the three countries were tracked during the study period, but only 287 were located, of whom 91 participated in the study. Of the 91 participants, 76 were being prescribed ART, and 15 had not started treatment. The main unintentional reason for missing clinic visits was a conflicting demand on the patients' time, which was often unexpected and for complex reasons, such as caring for a dying relative, going to a funeral, or traveling to work. These reasons were often transient and changed over time. Intentional reasons were often related to dissatisfaction with the care received at the clinic, especially “the harsh treatment” they received from health workers, which typically referred to behavior perceived by patients to be rude. For example, participants reported being spoken to “roughly” or feeling that the clinic staff “didn't care.” Such behavior made patients feel hurt, humiliated, and angry, and reluctant to return to the clinic. The researchers found that, overall, disengagement from care appeared to be a process through which missed visits and subsequent reluctance to return over time eroded patients' sense of connectedness to care.

**What Do These Findings Mean?:**

Absences from care will be inevitable over a lifetime course of treatment for HIV/AIDS. These findings indicate that absences may be unintentional as well as intentional, and that the reasons are complex and can change over time. Initial reasons for missing may disappear, leaving patients free, but reluctant, to return to care. Reasons for reluctance include shame at having been absent and the anticipation of a negative response to return from care providers. Patient education for ART initiation in sub-Saharan Africa often includes stern warnings about the lifelong commitment beginning ART represents. Paradoxically, educational efforts intended to maximize the benefits of ART for patients may be driving some away from care. Therefore, efforts to prevent missed clinic visits coupled with strategies to minimize any obstacles to coming back to care are necessary to keep patients' missed visits from turning into long-term disengagement from treatment.

**Additional Information:**

Please access these websites via the online version of this summary at http://dx.doi.org/10.1371/journal.pmed.1001369.

- This study is further discussed in a *PLOS Medicine* Perspective by Edward Mills
- The World Health Organization has the latest data on access to ART
- The US National Institute of Allergy and Infectious Diseases has more information on types of ART, and also on adherence to treatment
- The US Centers for Disease Control and Prevention has some information about interventions to help improve adherence

## Introduction

The rollout of antiretroviral therapy (ART) in sub-Saharan Africa has brought lifesaving treatment to millions of HIV-infected individuals. To continue to benefit from therapy, however, patients must remain in care. Ensuring long-term retention in care and treatment for HIV/AIDS has proven challenging in resource-scarce settings [1]–[5]. Tracking studies in which patients lost to follow-up are sought in the community to ascertain outcomes suggest that 20%–80% of patients “lost” but alive are no longer in care [6],[7]. Previous research by Geng et al. [8] using tracking data to estimate outcomes for a large clinic population indicated that approximately one in six patients taking ART were not in care after 2 y. Understanding disengagement from care is essential to improving retention and, thus, long-term outcomes of treatment.

The existing literature on barriers to ART adherence sheds considerable light on the retention problem. Since adherence means not only following dosing regimens but also being able to refill prescriptions, and since prescriptions are refilled as part of routine follow-up visits in Africa, barriers to adherence and retention overlap. Travel distance to clinic sites and associated costs, stigma and fear of disclosure, competing demands for scarce resources, religious and cultural beliefs, and unanticipated obligations and events (e.g., attending a family funeral) are key adherence barriers identified through previous research that also bear upon retention [9]–[14]. Motivation stemming from improved health following ART initiation, material and emotional support from others, and the promise of increased longevity are overlapping facilitators [15],[16].

As retention research develops, a number of patterns are emerging to suggest factors shaping disengagement. Disengagement rates appear particularly high for patients who have not yet initiated ART [17]–[23], and lower in decentralized care sites [24]–[27]. There is some evidence that younger patients, men, pregnant women, and individuals who have not disclosed their HIV status disappear from care more frequently [23],[28]–[32].

A wide variety of contributors to missed visits have been cited in patients' own accounts. Patient-reported barriers include travel distance to clinic, and associated time and expense; traveling/being away from home on the visit date; fear of disclosure; stigma; dissatisfaction with care; improved health; discouragement and the desire to “give up”; “return to normal life”; and a relatively low perception of risk associated with HIV [33]–[40]. Psychological facilitators have also been suggested, among them motivation, high perceived risk associated with HIV, and a sense of self-efficacy (feeling capable of meeting treatment requirements) [35],[41]. The number of studies with data on reasons for missed visits collected from patients has grown rapidly in recent years. In most cases, these data are reported simply as lists of reasons patient participants have provided, stopping short of deeper analysis.

For a more integrated, interpretive approach, we may look to the “meta-ethnography” by Merten et al. [40], which reviewed existing qualitative studies and integrated data to propose an explanatory “line of argument”. Drawing inductively upon results from 31 primary studies, the authors highlighted the importance of social processes in mediating ART adherence and retention in HIV care in Africa. Patients' “social integrity” and “social careers”—both of which refer to negotiation of social relationships in the context of ART treatment—were offered as concepts to clarify the social processes involved.

The goals of our research are also explanatory. Our conceptual starting point is a social contextual explanation of ART adherence in Africa centering on patients' sense of social responsibility as a motivating factor. This sense of responsibility is seen as a reciprocal response to material and emotional help from family, treatment supporters, health care providers, and others without which continued adherence to ART and to clinic follow-up appointments would not be possible. As recipients of ongoing adherence help, individuals taking ART feel a responsibility to helpers to succeed at therapy. Success thus becomes both a social responsibility and a means of ensuring help continues to be available into the future [42].

This qualitative, patient-centered research aimed to help explain missed clinic visits and subsequent disengagement from HIV/AIDS treatment and care in sub-Saharan Africa. The study took place in three African countries—Nigeria, Tanzania, and Uganda—and had two objectives. First, we set out to inductively identify and characterize in depth reasons for missed visits from the perspectives of patients. Second, we worked to assemble identified reasons into a broader explanation of missed visits and disengagement from care.

## Methods

### Country Settings and Clinical Study Sites

Tanzania, Uganda, and Nigeria represent diverse geographic and cultural settings. However, all three countries have generalized HIV epidemics in which women are disproportionately affected [43]. Characteristics of the epidemics vary across countries, but all three have seen prevalence rates drop with global scale-up of free ART. Huge health gains have resulted from the scale-up effort, yet more than half of eligible adults remain without access to therapy. Recent estimates place the number of adults living with HIV/AIDS at 1–1.5 million in Tanzania and Uganda, and at 3.3 million in Nigeria (see Table 1). The difference in numbers reflects population differences, at least in part. At approximately 160 million, Nigeria's population is the largest of all African countries. The number of adults living with HIV/AIDS in Nigeria is reported to be the third highest in the world [44].

**Table 1:** Characteristics of country settings [63]–[74].

Characteristic	Country		
	Nigeria	Uganda	Tanzania
Population	158,423,000 (2010)	33,425,000 (2010)	44,841,000 (2010)
HIV prevalence before ART scale-up	4.5% (1998)	8.3% (1999)	8.0% (1998)
Year free ART became available	2006	2004	2004
HIV prevalence after ART scale-up	3.6% [3.3%–4%] (2009)	6.5% [5.9%–6.9%] (2009)	5.6% [5.3%–6.1%] (2009)
Number of adults on ART	359,200 (2010)	248,200 (2010)	258,100 (2010)
Percent eligible receiving ART	31.0% (2009)	47.0% (2011)	50.0% (2010)
Number of adults living with HIV	3.3 million [2.9–3.6 million] (2009)	1.2 million [1.1–1.3 million] (2009)	1.4 million [1.3–1.5 million] (2009)

95% CIs given in brackets; year given in parentheses.

Participating clinical study sites were the Jos University Teaching Hospital (JUTH) AIDS Prevention Initiative in Nigeria HIV/AIDS Clinic (APIN Clinic) in Jos, Nigeria; the Mwananyamala Hospital HIV/AIDS Care and Treatment Center in Dar es Salaam, Tanzania; and the Mbarara Regional Referral Hospital Immune Suppression Syndrome Clinic (ISS Clinic) in Mbarara, Uganda. All three are large clinics providing comprehensive treatment, care, and support services for patients with HIV/AIDS. Physicians, nurses, counselors, and pharmacists provide services to patients, supported by technical, administrative, and ancillary staff. The total number of direct care staff ranges widely across the sites, from fewer than 30 to more than 70 (full- and part-time). The JUTH APIN Clinic serves the city of Jos, with a population of approximately 1.5 million, and the surrounding central plateau region of Nigeria. Mwananyamala Hospital HIV/AIDS Care and Treatment Center serves the Kinondoni Municipality of Dar es Salaam, Tanzania's largest city, numbering about 4 million people. The ISS Clinic serves the Mbarara District of southwest Uganda and parts of neighboring districts (approximately 3 million people). Additional information about participating clinical study sites appears in Table 2.

**Table 2:** Characteristics of participating study sites.

Characteristic	APIN Clinic (Jos, Nigeria)	Mwananyamala Hospital HIV/AIDS Care and Treatment Center (Dar es Salaam, Tanzania)	ISS Clinic (Mbarara, Uganda)
Geographic setting	Urban	Urban	Peri-urban and rural
Size of population served	22 million	4 million	3 million
Year opened	2002	2004	1998
Total adults enrolled in HIV/AIDS care	17,139	16,107	21,149
Total adults currently followed	10,532	5,478	8,808
Adults currently followed on ART	8,854	3,930	7,140

### Patient Tracking for Research and Clinical Care

With the identification of loss to follow-up as a threat to the long-term success of ART scale-up in Africa, patient tracking has been increasingly relied upon by researchers as a means of obtaining information on patients who have missed clinic visits [7],[8],[37],[38]. At the same time, local treatment program planners and health care practitioners have independently developed patient tracking as part of HIV clinical care. For clinical purposes, tracking is used to encourage retention and to contact patients who need to be seen outside of regular appointment schedules. Clinical trackers are trained health care workers or volunteers. Permission for tracking is requested from patients at enrollment in care. In-person patient tracking is a culturally meaningful clinical strategy for making contact with patients in the absence of highly developed telecommunications systems. As mobile phones become more widely available, telephone tracking is replacing the in-person approach.

Clinical trackers travel outside clinics to locate patients in the community. From lists of patients to be tracked, trackers organize itineraries for in-person tracking and lists of individuals to be followed up by telephone, then attempt to make contact. The tracking programs at the three clinical sites participating in this study differed in size, mode of transport, reliance on phone versus in-person tracking, and distance traveled to locate patients. Differences reflected distinctions in clinic policy and/or local practice. Trackers at all three sites were paid and received training in HIV treatment, ethical practice, and tracking procedures.

### Sampling and Recruitment

The sample for this study was generated using clinic tracking lists. The lists identified adult patients aged 18 y and older who were (1) enrolled in care, (2) had missed a regularly scheduled follow-up visit, and (3) had not subsequently returned to the clinic for 3 mo or more. A 3-mo period of absence was chosen following the practices of participating clinics for identifying patients to be tracked. Lists of persons fulfilling these criteria were generated by data managers at the sites from clinic databases and forwarded to clinic trackers for follow-up. When an individual was contacted through clinic tracking, and after standard clinic tracking procedures had been completed, trackers briefly introduced the research using an institutional-review-board-approved script, and requested permission for follow-up by study research assistants. Individuals giving permission were then recontacted by research assistants, who described the study in detail, answered questions, and enrolled those who expressed interest in participating. Thus, individuals participating in the study met the following inclusion criteria: (1) were absent from the clinic for 3 mo or more, (2) were tracked and contacted by clinic trackers, (3) gave permission for additional follow-up by study research assistants, (4) were successfully recontacted by study research assistants, and (5) were able and willing to provide informed consent.

### Ethics

This study was approved by the Harvard Medical School Committee on Human Studies, Boston, Massachusetts; the JUTH Institutional Health Ethical Research Committee, Jos, Nigeria; the Muhimbili University of Health and Allied Sciences Senate Research and Publications Committee, Dar es Salaam, Tanzania; the Mbarara University of Science and Technology Institutional Review Committee, Mbarara, Uganda; and the Uganda National Council on Science and Technology, Kampala, Uganda. All study participants provided written informed consent. Compensation in the form of cash and/or reimbursement for travel to interview locations was provided. Amounts and forms of compensation were determined in consultation with investigators and ethical review boards at participating sites.

### Data Collection

Data were collected from 15 January 2010 through 30 March 2012 using in-depth in-person interviews with enrolled patient participants. Consistent with an inductive approach, interviews were designed to be flexible, following a general, topic-oriented structure. Topics were selected to address the goals of the research and included the following: (1) experiences of care at the clinic, (2) experiences of tracking, and (3) circumstances of missed appointments. Interviews sought to elicit detailed accounts of actual experiences of interviewees from their points of view. They were conducted by trained research assistants in each of the three countries in local languages, and were audio-recorded with permission. They took place in private locations—at patients' homes or at other convenient places of their choosing—and lasted approximately one hour.

### Data Preparation

Upon completion of each interview, interviewers produced a complete transcript in English from local language audio-recordings. Each transcript was reviewed for quality by M. A. W. Interviewers made additions and corrections to transcripts as indicated by the quality review. The transcripts constituted the study data.

### Data Analysis

Analysis of data aimed to characterize patients' reasons for missing clinic visits and to represent them as descriptive categories. An inductive approach to concept development centering on category construction and informed by grounded theory methodology [45],[46] was used to carry out the analysis. Analysis began with repeated review of interview transcripts to identify sections of text illuminating reasons for missing clinic visits. These sections of text were displayed in matrices to reduce the data and facilitate identification of content similarities or patterns. Sections of text determined to be similar in content were grouped and assigned provisional labels to form an initial set of “reason categories.” The initial category set was then reviewed to confirm that labels accurately reflected corresponding sections of text. Labels were revised as necessary to strengthen correspondences and improve precision. Illustrative quotes were retrieved from interview transcripts. Multiple authors participated in this process. Disagreements and inconsistencies arising in the analytic process were resolved through discussion. Data from the three sites were analyzed separately.

Once the categories were formulated, they were examined interpretively for broader underlying dimensions and relationships to each other. This interpretive analysis allowed us to move beyond a listing of individual reasons for missing clinic appointments toward a larger, more integrated explanation, and to highlight previously underemphasized aspects of the problem. Categories were then further grouped to represent “unintentional” versus “intentional” reasons for missing appointments and were loosely arranged in time sequence. Of course, other interpretations are possible. Our goal was to advance thinking and research on disengagement from HIV care in sub-Saharan Africa by proposing new conceptualizations suggested by qualitative data.

## Results

### Outcomes of Tracking

Eight-hundred-ninety patients were tracked at the three sites as part of recruitment. Two-hundred-eighty-seven were located, and 91 ultimately took part in the study (see Table 3). Of the 91 participants, 76 were being prescribed ART; 15 had not initiated treatment. Length of time out of care varied widely across the sample of participants. Reasons for not participating in the study among located patients included the following: (1) refusal to give permission for follow-up contact for research, (2) denial of HIV-positive status to trackers, (3) fear of disclosure of HIV status as a result of research participation, and (4) preference for remaining uninvolved in any further HIV-focused activity (treatment or research). Of 603 patients tracked but not located, 211 (35%) were reported dead, 144 (24%) had moved, and 79 (13%) were traveling away from home at the time of tracking visits. No information could be obtained for the remainder.

**Table 3:** Study tracking activities and outcomes.

Site	Number of Trackers Participating	Tracking Period for Study Recruitment	Number of Patients Tracked	Number of Patients Located (Percent)	Number of Patients Enrolled
Jos, Nigeria	3	18 mo	254	140 (55%)	40
Dar es Salaam, Tanzania	19	6 mo	208	46 (22%)	20
Mbarara, Uganda	2	17 mo	428	101 (24%)	31

Of the 140 people located in Jos, 17 (12%) were tracked by home visit only (no phone). In Mbarara and Dar es Salaam, the large majority of people were tracked by home visit only.

### Characteristics of Interviewees

The average age of patient interviewees was 36 years. Almost 60% were women; 62% were Christian. On average, they had a little less than 10 y of education. Three-quarters of interviewees reported being employed. Median reported travel time to clinic was about 45 min. Nigerian participants had more years of schooling than Ugandan or Tanzanian participants, and were more likely to be employed than Tanzanian participants. Travel time to clinic was longest in Uganda. Characteristics of patient interviewees are detailed in Table 4.

**Table 4:** Patient participant interviewee characteristics.

Characteristic	All (*n* = 91)	Nigeria (*n* = 40)	Tanzania (*n* = 20)	Uganda (*n* = 31)
**Gender female (percent)**	59.3	62.5	55.0	58.1
**Age (years), mean (SD)**	35.9 (8.1)	34.9 (7.4)	39.3 (9.0)	35.0 (8.1)
**Religion (percent)**				
Christian	61.5	52.5	50.0	80.1
Muslim	38.5	47.5	50.0	19.4
**Education (years), mean (SD)**	9.8 (3.9)	11.9 (3.7)	9.1 (2.8)	7.6 (3.6)
**Employment (percent)**				
Employed	75.8	85.0	55.0	77.4
Unemployed	24.2	15.0	45.0	22.6
**Travel time to clinic (minutes), median (IQR)**	45 (30–120)	30 (30–60)	30 (30–52.5)	120 (60–180)

IQR, interquartile range; SD, standard deviation.

### Reasons for Missing Visits

Core terms used in the discourse on retention in HIV treatment and care reflect the evolution of research and thinking on the topic, while pointing to one or another potential source of retention problems. “Loss to follow-up” connotes clinics' difficulties in keeping track of patients, while patient-centered terms—the currently preferred “disengagement” or the African term “defaulter”—imply that missed visits result from patient choice. In contrast, it was the *unintentional* origins of missed visits that stood out among our study participants.

Interviewees continually confronted competing demands on their time. When demands stemmed from cultural and family obligations, or economic requirements—e.g., caring for a sick family member in a distant location, traveling for work, attending a relative's funeral in another town—they took precedence over keeping clinic appointments. The following interview excerpt illustrates this:

*I went to the village in December and when it was time to come back before my appointment of January 3, 2011, I was not able to come back because I lost a brother. A few days after losing my brother, I lost another relative and so I stayed in the village. I had planned to come back on January 1, since my clinic appointment was for January 3, but I did not come back.* [Female, age 27]

Plans were routinely upended by unexpected events, another source of unintentional absences. Accidents, vehicle breakdowns, and other travel delays meant that individuals who had traveled but planned to return before their clinic visit date were stranded away from home, as in the following case:

*What caused me not to come to the clinic was that I lost my father. When he died, I went to [name of town] for the burial and the money that I had taken with me ran out. And so I had to first stay there to make some more to facilitate my return. When I was able to return to my home, I failed to get the money for transport to the hospital and so I started working to be able to earn the amount enough to facilitate my transport fare.* [Male, age 40]

Encounters with violence were obstacles for some patients. Lack of support from family and community in the form of failure to provide transport funds or of active opposition to care led to unintentional absences for some interviewees. Errors—misunderstanding visit dates or forgetting an appointment—were another unintentional reason for missing.

Some missed visits were intentional. For example, patients might decide to stop attending a particular clinic as decentralization initiatives provided the option of receiving care closer to home. Patients offered an alternative to long, expensive travel for care transferred to local facilities. If transfers were not recorded at the initial care sites, those patients were categorized as “missing.”

When patients decided to stop keeping clinic appointments, the reason could also be dissatisfaction with care. Interviewees reported objections to clinic policy (e.g., insistence on adherence to certain days and times of appointments) or some aspect of care organization (e.g., clinic schedules that made for long waiting times). Interviewees also complained of harsh treatment by providers.

Harsh treatment typically referred to behavior perceived by patients to be rude and/or rejecting. For example, interviewees reported being spoken to “roughly” or feeling that the clinic staff “didn't care.” “Shouting” and “bad language” were cited. At one site, references were made to threats that missed visits would result in being dropped from care. Experiences of harsh treatment left patients feeling hurt, angry, and humiliated, as the following quotes illustrate:

*I felt humiliated. I felt very bad. After receiving the services I was full of pain. After that incident I didn't go back to the hospital. I decided to leave everything and opt for fruits and food…and until now I am not taking it [ART].* [Male, age 60]

*Like my last clinic visit, it became too much. They really looked like they didn't care. [Interviewer: Why do you say they looked like they didn't care?] When people come to the clinic and go to their [consulting] rooms, they don't care about them. There are many patient files piled on their desks but they just sit there. Or sometimes, they come, look at the files and then just go their own way. Or sometimes when you enter the doctor's room, they start conversing and talking about their own things while you just sit there and wait for them to finish.* [Female, age 27]

*…the first day I came, I didn't know I had to drop my card—hand card—by the door. So I came in and sat down where my friend said. The nurse there was so harsh that I didn't drop my card. She said all nasty things to me and at a point I said, “Amen! Let me just go.” That was the first day; that was the first experience. I felt it was not a place to be. It was like I should just leave the hospital immediately.* [Female, age 35]

*They [staff] told me, “You are late.” Now, there are problems people face. I don't know how they perceive it but for me this thing is very difficult. Attending clinic every month is very difficult, because you have to leave your work, sometimes report [to work] late—reasons like these. Everyone has problems. They are supposed to solve these problems with love, not harshly like they do. Until people are afraid of their words—abusive words. They behave as if we are there to beg for meds. It's our right to get the meds.* [Female, age 34]

Examined closely, absences are revealed to be less the simple result of one or another “reason” taken individually, and more the product of complex chains of events. To identify only the initial, “surface” reason is to provide an incomplete, sometimes inaccurate, account. For a full understanding, one must follow the events in the chain. An example is the frequently cited “problem of transport.” Transport problems often gloss a much more complicated set of circumstances. In the following instance, for example, an interviewee who began his explanation of missed visits by citing a “transport problem,” went on to reveal more about what that problem led to and meant:

*We fail to get vehicles sometimes. And when you go to look for money for [a motorcycle taxi] you find you do not have it. So when you miss your appointment and go to clinic on another day, [the provider] starts quarreling with you about not having come on the appointed day. And when you tell that person you got problems, he tells you, “You should spend the night on the road.” How can I spend the night on the road? Here I am, having failed to get money for taking me to the hospital and then I'm supposed to get money to spend the night somewhere and feed myself? These are some of the problems I have in going to the clinic.* [Male, age 56]

This patient became discouraged and abandoned care—not so much due to transport difficulties as to the lack of understanding and accommodation of these difficulties by staff. When located for this study, he had not been seen for care for 2 y.

In addition to being complex, many obstacles to missed visits are also transitory. They end, dissipate, or cease to be obstacles as circumstances change over time. Recognizing this raises the question, if initial obstacles no longer block clinic attendance, why don't patients who miss visits then return? A strong reluctance to return for care after an absence emerged from the qualitative data.

*I was scared to come back to the clinic since a lot of time had already passed and I might come back and they chase me away.* [Male, age 40]

*I was scared of coming back and them telling me that they will not accept me because I didn't come when they told me to. I was wondering whether they would accept me or not or whether they would scold me.* [Female, age 35]

*I was afraid to go back because I didn't have my card and I stopped meds for a long time. I was afraid the nurses would yell at me.* [Female, age 35]

### Reluctance to Return

Two reasons for this reluctance stand out. The first centers on a sense of shame articulated by interviewees, who felt a strong sense of the responsibility that comes with starting ART. Having gained access to a valuable resource in a resource-scarce environment, and having been extensively “educated” as to the adherence commitment access to that resource entails, patients who miss clinic visits seemed to see themselves as having failed to live up to their responsibilities when they missed visits and experienced a treatment lapse. The following quotes illustrate this:

*When they accept us and open our files, they tell us this—that when you start, you should not stop. And if you know you will play with it, then do not start. So it was because I know I was wrong. That is why I said I will not be able to return.* [Female, age 25]

*In fact, when [the tracker] called me, I felt a sense of guilt. This is my health. I am supposed to be worrying about my health, not somebody else worrying about my health—you know?* [Male, age 46]

Apprehension at anticipated negative responses from care providers is a second explanation suggested by the data for reluctance to return. Over time, the distress at perceived poor treatment prompting patients to miss visits evolves into anticipation of the painful encounters they see awaiting them upon return. Interviewees articulated a fear of being “yelled at,” “abused,” and/or “chased” from the clinic should they attempt to resume treatment after an absence. Rather than face this, they simply stayed away.

*I was afraid to go back because I had stopped meds for a long time and I didn't have my [clinic identity] card. I was afraid the nurses would yell at me….* [Female, age 35]

*I did not come back on the date I was given. When the date for me to go back to the clinic had passed, I feared I would be abused if I went back past my appointment date.* [Female, age 20]

*I was scared to come back to the clinic since a lot of time had already passed and I might come back and they chase me away.* [Female, age 20]

*The way she told me, “Why did you miss clinic…despite what happened, you should have come to the clinic and met other people.” I told her no, I didn't want to show up because what happened to me [at the clinic] was still in my heart. If I was treated nicely the situation would have been different; since I was treated badly I saw the treatment was meaningless.* [Male, age 60]

*[Interviewer: The situation of stopping care or treatment at the clinic, how did that make you feel?] It made me feel bad to see that I had to go to get meds. Looking at how badly the provider spoke to me, I asked him how they would be benefiting from my death? I said to myself, “Let me leave these things. If I am to die let me do that—die.”* [Male, age 56]

**Figure 1:**
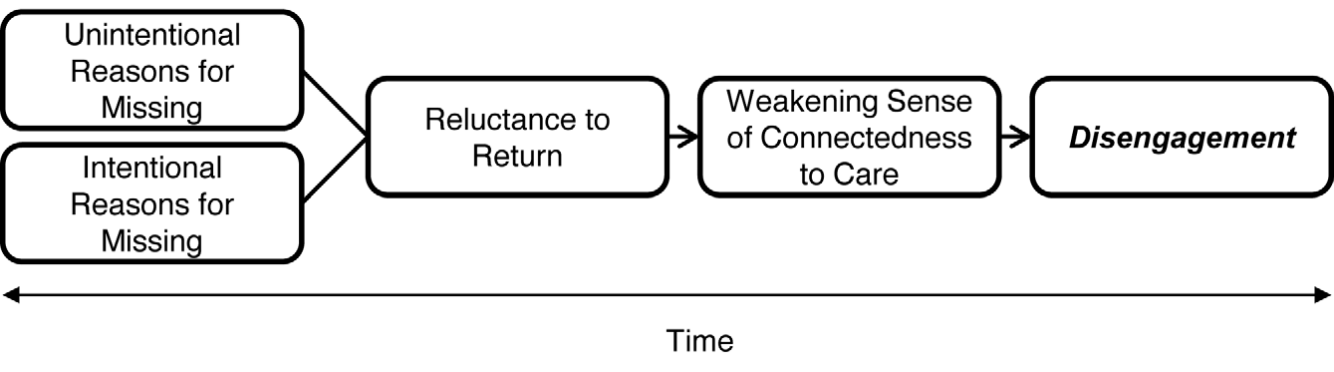
How missed visits may become disengagement from HIV/AIDS treatment and care in Africa.

Reluctance to return, whether originating in shame at having failed to fulfill a responsibility or apprehension about the anticipated negative response of providers, signals the erosion of a subjective sense of connectedness to the idea, processes, and, for some, the specific site of care. We propose it is this sense of connectedness, together with family and community support, that fuels patients' motivation to consistently overcome economic and other obstacles to keeping clinic appointments. The lifelong commitment patients understand themselves to be making in initiating ART, a sense of caring and expectation of success from providers, and solidarity with fellow patients all contribute. The data point to patients' subjective sense of connectedness to care as a significant, and to date underappreciated, antecedent of retention (see Figure 1).

## Discussion

This qualitative study reveals the importance of recognizing the unintentional origins of many missed visits, the often hidden complexity of reasons for missing, and the ways in which reasons change over time. A prospective approach to the qualitative data revealed both the transitory nature of some obstacles to clinic attendance and a trajectory whereby initial obstacles develop into reluctance to return. In highlighting these broader dimensions and identifying “reluctance to return” as an analytic construct, our aim is to move away from a narrow focus on individual barriers toward a larger and more integrated explanation.

The prominence of reluctance to return in interviewee accounts of absences from care signals a potential caution for providers overseeing initiation of ART. Patient education in clinical settings for ART initiation in Africa has featured stern warnings about the lifelong commitment treatment represents, the meaning and importance of medication adherence, and the dire consequences of lapses. Patients' feelings of shame at missing visits and fear of clinic staff's negative response to absences may indicate just how seriously they have taken these warnings. Educational efforts intended to maximize the benefits of ART for patients may paradoxically be driving some away from care.

How shall we understand the patient complaints of harsh treatment by staff seen in accounts of reluctance to return? The quality of relationships between patients and health care providers in poor countries has received relatively little research attention. However, the available literature does clearly indicate that friction between the two groups, and reports of “abuse” by clinical staff, are neither specific to HIV/AIDS care nor limited to the sites and countries represented here [47]–[53]. Analyses shedding light on the origins of friction are scarce, but suggest as contributing factors the overwhelming numbers of patients and the emotional strain of HIV/AIDS care provision [54]–[57], perceived threats to professional identity [47],[52], and conflicting priorities of patients and staff [51].

Qualitative research in medicine and public health intentionally aims for in-depth analysis rather than generalizable results. Nevertheless, single site qualitative studies may reflect limitations in resources more than dictates of research design. This study was carried out at three HIV treatment sites in three sub-Saharan African countries. Results reflect commonalities and contrasts across the three patient participant groups. In all three, visits are missed both unintentionally and by design, and evolve into reluctance to return. The specific forms this pattern takes vary, however. The availability of data from three sites in this study offer the advantage of a broader base for analysis, but do not allow for formal cross-site comparison, as the qualitative samples were not designed to be representative.

The recent shift in emphasis from increasing access to HIV treatment to enhancing retention in care has led to a number of suggestions and changes aimed at reducing absences. Some of these are structural, e.g., reorganizations of services to make clinic attendance more convenient and efficient (decentralization, reduced waiting times, reduced visit frequency) [58]. Others feature one or another form of community-based support [59],[60]. Returning patients to care following an absence has received less attention as an intervention objective overall.

Yet recent research confirms substantial numbers of lost patients are alive and can be located [7],[8],[38],[61],[62]. This study calls attention to reluctance to return as a retention barrier. It follows that interventions designed to address and resolve reluctance might inspire “missing” patients to return to care [61]. To target return to care (rather than prevention of missed visits) would be to bring a harm reduction approach to the retention problem, and to recognize that, while not optimal, absences will be unavoidable over a lifetime of treatment. A harm reduction approach would seek to reduce barriers to re-engagement.

The process through which unintentional and intentional missed visits evolve into a weakened sense of connectedness, reluctance to return, and, ultimately, disengagement from care points to an underlying exchange-based relationship between health care providers and HIV/AIDS patients receiving care and ART. In return for access to lifesaving treatment and care, patients are expected to, and agree to, reciprocate with adherence to both medication and clinic appointments. The commitment entails a moral obligation; missed visits are thus a moral failing. Understanding the moral dimensions of the treatment relationship makes sense of patients' shame, justifies “scolding” by providers, and explains the common usage of the term “defaulter” (i.e., someone who fails to repay a debt) to refer to patients who miss clinic appointments in sub-Saharan Africa.

This study has a number of limitations. First, the study is based on a convenience sample of patients who could be located through tracking. Reasons for missed visits among persons who could not be located are not represented. Social desirability bias and recall bias may have also resulted in some reasons for missing visits not appearing in the data. Neither reasons for returning to care following an absence nor differences across the clinical sites is examined. A limitation of this analysis qualitatively is that it reflects only a single perspective: the patient's perspective. In a more comprehensive qualitative examination, multiple perspectives on the retention problem would be represented.

### Conclusion

International scale-up of treatment and care for HIV/AIDS has been a resounding public health success. To realize full benefit from the initiative, high rates of long-term retention are essential. Retention research focusing on Africa has shed considerable light on the dimensions of the problem and on reasons for absences from care as directly reported by patients.

This paper offers a broader and more complete explanation of disengagement and a new explanatory construct: reluctance to return. To focus on return is to acknowledge the inevitability of absences over a lifetime course of treatment for HIV/AIDS. Efforts to prevent missed clinic visits complemented by moves to minimize barriers to re-entry into care are more likely than either alone to keep missed visits from turning into long-term disengagement.

## Abbreviations

APIN Clinic: AIDS Prevention Initiative in Nigeria HIV/AIDS Clinic
ART: antiretroviral therapy
ISS Clinic: Mbarara Regional Referral Hospital Immune Suppression Syndrome Clinic
JUTH: Jos University Teaching Hospital
